# HIF‐1α modulates pancreatic cancer ECM proteins via the TGF‐β1/Smad signaling pathway introduction

**DOI:** 10.3389/fonc.2025.1564655

**Published:** 2025-05-08

**Authors:** Houle Guo, Zhanxue Zhao, Linxun Liu

**Affiliations:** ^1^ Suzhou Medical College of Soochow University, Suzhou, China; ^2^ Hepatological Surgery Department, Qinghai Provincial People’s Hospital, Xining, Qinghai, China

**Keywords:** transforming growth factor beta 1, hypoxia-inducible factor-1 alpha, extracellular matrix, Smad signaling pathway, pancreatic cancer

## Abstract

**Introduction:**

Pancreatic cancer is characterized by its aggressive nature and poor prognosis, ranking among the most lethal malignancies. The tumor microenvironment, particularly the extracellular matrix (ECM), plays a crucial role in cancer progression. This study investigated the relationship between hypoxia-inducible factor-1α (HIF-1α) and transforming growth factor-β1 (TGF-β1) in regulating ECM protein expression in pancreatic cancer.

**Methods:**

PANC-1 cells were cultured under both normoxic and hypoxic conditions. Pharmacological inhibition of HIF-1α and TGF-β1, as well as TGF-β1 stimulation, were employed to evaluate ECM protein expression. HIF-1α knockdown experiments and co-immunoprecipitation were performed to assess molecular interactions. Clinical specimens were analyzed for HIF-1α and TGF-β1 expression.

**Results:**

HIF-1α was found to modulate ECM protein expression through the TGF-β1/Smad signaling pathway. Pharmacological inhibition of either HIF-1α or TGF-β1 significantly decreased the expression of ECM proteins, while TGF-β1 stimulation enhanced their production. HIF-1α knockdown abolished TGF-β1-induced ECM protein expression, indicating that HIF-1α is essential for TGF-β1-mediated ECM regulation. Co-immunoprecipitation experiments revealed a physical interaction between HIF-1α and TGF-β1. Clinical specimens showed significantly elevated expression of both HIF-1α and TGF-β1 in pancreatic cancer tissues compared to adjacent normal tissues, correlating with advanced disease stages.

**Discussion:**

These findings elucidate a novel mechanism where HIF-1α and TGF-β1 cooperatively regulate ECM production in pancreatic cancer, providing potential therapeutic targets for intervention.

## Introduction

Pancreatic cancer remains one of the most lethal malignancies worldwide, characterized by rapid progression and poor clinical outcomes (1). Despite advances in therapeutic strategies, including surgery, chemotherapy, and targeted therapies, the five-year survival rate remains below 10% (2). This stark reality underscores the urgent need to elucidate the molecular mechanisms driving pancreatic cancer progression and to identify novel therapeutic targets.

The tumor microenvironment (TME) plays a pivotal role in cancer progression, with the extracellular matrix (ECM) serving as a critical component (3). The ECM, a complex network of proteins and glycosaminoglycans, provides structural and biochemical support to surrounding cells (4). In pancreatic cancer, ECM alterations significantly contribute to tumor growth, invasion, and metastasis (5). Notably, the dense fibrotic stroma characteristic of pancreatic cancer not only supports tumor progression but also creates a formidable barrier to drug delivery, contributing to therapeutic resistance (6, 7).

Hypoxia-inducible factor-1α (HIF-1α), a key transcription factor activated under hypoxic conditions—a hallmark of solid tumors, including pancreatic cancer (8)—regulates genes involved in angiogenesis, metabolism, and cell survival, enabling cellular adaptation to low oxygen environments (9). Similarly, transforming growth factor-β1 (TGF-β1), a multifunctional cytokine, regulates ECM production, cell proliferation, and differentiation (10). In pancreatic cancer, TGF-β1 promotes tumor progression and enhances ECM deposition, driving the characteristic desmoplastic reaction (11).

While the molecular mechanisms of ECM remodeling have been extensively studied, the synergistic interplay between hypoxia and pro-fibrotic signaling pathways remains incompletely understood. The hypoxic tumor microenvironment in pancreatic cancer stabilizes HIF-1α to drive pro-survival gene expression; however, whether HIF-1α directly regulates ECM components, such as collagens, remains controversial. Previous studies suggest that HIF-1α activates pro-fibrotic factors (e.g., connective tissue growth factor, CTGF), but its relationship with the TGF-β1 pathway has not been systematically explored in pancreatic cancer (12, 13). TGF-β1 is a master regulator of ECM deposition, directly upregulating collagen synthesis via the Smad signaling pathway (14). Nevertheless, whether the pro-fibrotic effects of TGF-β1 under hypoxia depend on HIF-1α remains unclear. Emerging evidence indicates that HIF-1α may enhance TGF-β1/Smad pathway activity through non-canonical routes (e.g., PI3K/Akt) (15), yet the role of this mechanism in pancreatic cancer ECM regulation remains uncharacterized. Furthermore, physical interactions between HIF-1α and Smad proteins have been reported in other cancer types (16). Based on these findings, we hypothesize that the hypoxic microenvironment in pancreatic cancer activates the TGF-β1/Smad signaling pathway via HIF-1α, thereby synergistically promoting the expression of ECM proteins (e.g., collagen I/III/V) and exacerbating tumor invasion and stromal fibrosis.

In this study, we investigated the interplay between HIF-1α and TGF-β1 in regulating ECM protein expression through the Smad signaling pathway in pancreatic cancer. Using *in vitro* models and clinical specimens, we examined how these factors cooperatively influence ECM production under normoxic and hypoxic conditions. Our findings reveal a novel mechanism by which HIF-1α modulates pancreatic cancer ECM via the TGF-β1/Smad axis, offering potential therapeutic strategies to target the dense stromal component of this malignancy.

## Materials and methods

### Clinical sample collection

Pancreatic cancer tissues and adjacent normal tissues (≥1 cm from tumor margin) were collected from 30 patients who underwent surgical resection at Qinghai Provincial People’s Hospital between January 2020 and December 2021. The study was approved by the Hospital Ethics Committee (approval number: QPH-2020-015), and written informed consent was obtained from all patients. No patients received preoperative chemotherapy or radiotherapy. Clinical staging followed the AJCC 8th Edition Guidelines for pancreatic cancer (See **Table 1**).

**Table 1:** Clinical staging of pancreatic cancer.

TNM staging	
0 stage	Tis N0 M0
IA stage	T1 N0 M0
IB stage	T2 N0 M0
IA stage	T3 N0 M0
IIB stage	T1-3 N1 M0
III stage	Stage T4, any N, M0
IV stage	Stage any T, any N, M1

### Cell culture and treatments

PANC-1 human pancreatic cancer cells (ATCC^®^ CRL-1469™) were maintained in DMEM (Gibco, 11995065) supplemented with 10% FBS (Gibco, 16140071) and 1% penicillin/streptomycin at 37°C in 5% CO2. For hypoxic conditions, cells were cultured in a hypoxia chamber (1% O2, 5% CO2, 94% N2) for 24 hours. Treatments included:

- HIF-1α inhibitor PX-478 (Selleck, S7612, 25 µM) Treatment duration was 24 hours.
- TGF-β1receptor inhibitor LY2157299 (Selleck, S2230, 10 µM) Treatment duration was 24 hours.
- Recombinant human TGF-β1 (R&D Systems, 240-B, 10 ng/mL) Treatment duration was 24 hours unless otherwise specified.

### siRNA transfection

HIF-1α siRNA (5′-CCACCACUGAUGAAUUAAATT-3′) and control siRNA were synthesized by GenePharma. Transfection was performed using Lipofectamine™ RNAiMAX (Invitrogen, #13778150) following the manufacturer’s protocol. PANC-1 cells were seeded into 24-well plates at a density of 5 × 10^4^ cells/well and cultured in DMEM/F12 complete medium supplemented with 10% fetal bovine serum (FBS) and 1% penicillin/streptomycin at 37°C under 5% CO_2_ until reaching 60–70% confluency. For each well, 25 pmol of HIF-1α siRNA or control siRNA was diluted in 5 μL of Opti-MEM^®^ Reduced Serum Medium. Separately, 0.75 μL of Lipofectamine™ RNAiMAX was mixed with 5 μL of Opti-MEM^®^ and incubated at room temperature for 5 minutes. The diluted siRNA and Lipofectamine™ complexes were then gently combined and incubated for an additional 15 minutes at room temperature to allow complex formation. The original medium in the wells was aspirated and replaced with 500 μL of fresh Opti-MEM^®^. The siRNA-lipid complexes were added dropwise to each well, followed by gentle swirling to ensure even distribution. After 6 hours of transfection, the medium was replaced with complete DMEM/F12, and cells were further cultured until the designated time points for analysis.

### Western blotting

When cells reached approximately 80% confluency, they were scraped and lysed using 300 μL of RIPA lysis buffer (Thermo Scientific, #89900) supplemented with protease inhibitors. The collected cell suspension was incubated on ice for 15 minutes. Lysates were centrifuged at 12,000 rpm for 20 minutes at 4°C to pellet debris, and the supernatant was collected. Subsequently, 75 μL of 5× loading buffer was added to the supernatant, and the mixture was boiled at 95°C for 15 minutes. Total proteins were separated via 12% SDS-polyacrylamide gel electrophoresis (SDS-PAGE) and transferred onto PVDF membranes. Membranes were blocked with 5% non-fat milk for 1 hour at room temperature. Primary antibodies were diluted in blocking buffer and incubated with the membranes overnight at 4°C, followed by incubation with horseradish peroxidase (HRP)-conjugated secondary antibodies for 2 hours at room temperature. Protein bands were visualized using a molecular imaging system (VersaDoc MP 4000, Bio-Rad).

Primary antibodies included:

- HIF-1α (Cell Signaling Technology, #36169, 1:1000)
- TGF-β1 (Abcam, ab215715, 1:1000)
- Collagen I (Abcam, ab34710, 1:1000)
- Collagen III (Abcam, ab7778, 1:1000)
- Collagen V (Abcam, ab7046, 1:1000)
- Smad2 (Cell Signaling Technology, #5339, 1:1000)
- Smad3 (Cell Signaling Technology, #9523, 1:1000)
- Smad4 (Cell Signaling Technology, #38454, 1:1000)
- β-actin (Sigma-Aldrich, A5441, 1:5000).

### Co-immunoprecipitation (Co-IP)

Cell lysates were incubated with anti-HIF-1α antibodies overnight at 4°C. Protein A/G magnetic beads (Thermo Scientific, #88802) were then added, and the mixture was incubated for an additional 2 hours at 4°C with gentle rotation. Immune complexes were pelleted by centrifugation, washed three times with ice-cold PBS, and resuspended in 1× Laemmli buffer. Western blot experiments using anti-TGF-β and Smad3 antibodies to detect immunoprecipitated proteins.

### Immunohistochemistry

The tissues were fixed in cold 4% ParaFormAldehyde (PFA) and embedded into paraffin blocks. After carrying out the de-paraffinization, dehydration, antigen retrieval, and blocking processes, the tissue sections were incubated with antibodies overnight at 4°C. Then, all the tissue sections were incubated with the biotinylated goat anti-rabbit IgG for 20 mins at RT, followed by streptavidin-horseradish peroxidase for 30 mins. Finally, diaminobenzidine-H2O2 and hematoxylin were used for staining the tissues.

Expression levels were scored by two independent pathologists blinded to clinical data. Scoring criteria:

- Intensity: 0 (negative), 1 (weak), 2 (moderate), 3 (strong)
- Percentage of positive cells: 0 (0%), 1 (1-25%), 2 (26-50%), 3 (51-75%), 4 (76-100%) Final score = intensity × percentage. Scores ≥6 were considered high expression

### Transwell migration and invasion assay

Migration and invasion experiments were performed using Boyden chambers consisting of Transwell membrane filter inserts (Cat # 3422, Corning Costar). In brief, 5 × 104 cells were seeded into each 24-well Transwell chamber (8 μm pore size) for migration assay, or into chambers coated with Matrigel for the invasion assay, in complete medium with 10% FBS. Migration and invasion cultural periods were 24 h and 48 h, respectively. Cells that did not penetrate the filter were wiped off, and cells on the lower surface of the filter were stained with 0.4% crystal violet. The numbers of migrating or invading cells were counted under a light microscope from five fields in a single chamber of three samples (mean ± SE).

### Statistical analysis

Data are presented as mean ± SD from at least three independent experiments. Statistical analysis was performed using SPSS 26.0. Student’s t-test or one-way ANOVA with Tukey’s *post-hoc* test was used for comparisons. P < 0.05 was considered statistically significant.

## Results

### Elevated expression of HIF-1α and TGF-β1 in pancreatic cancer tissues

**Figure 1:**
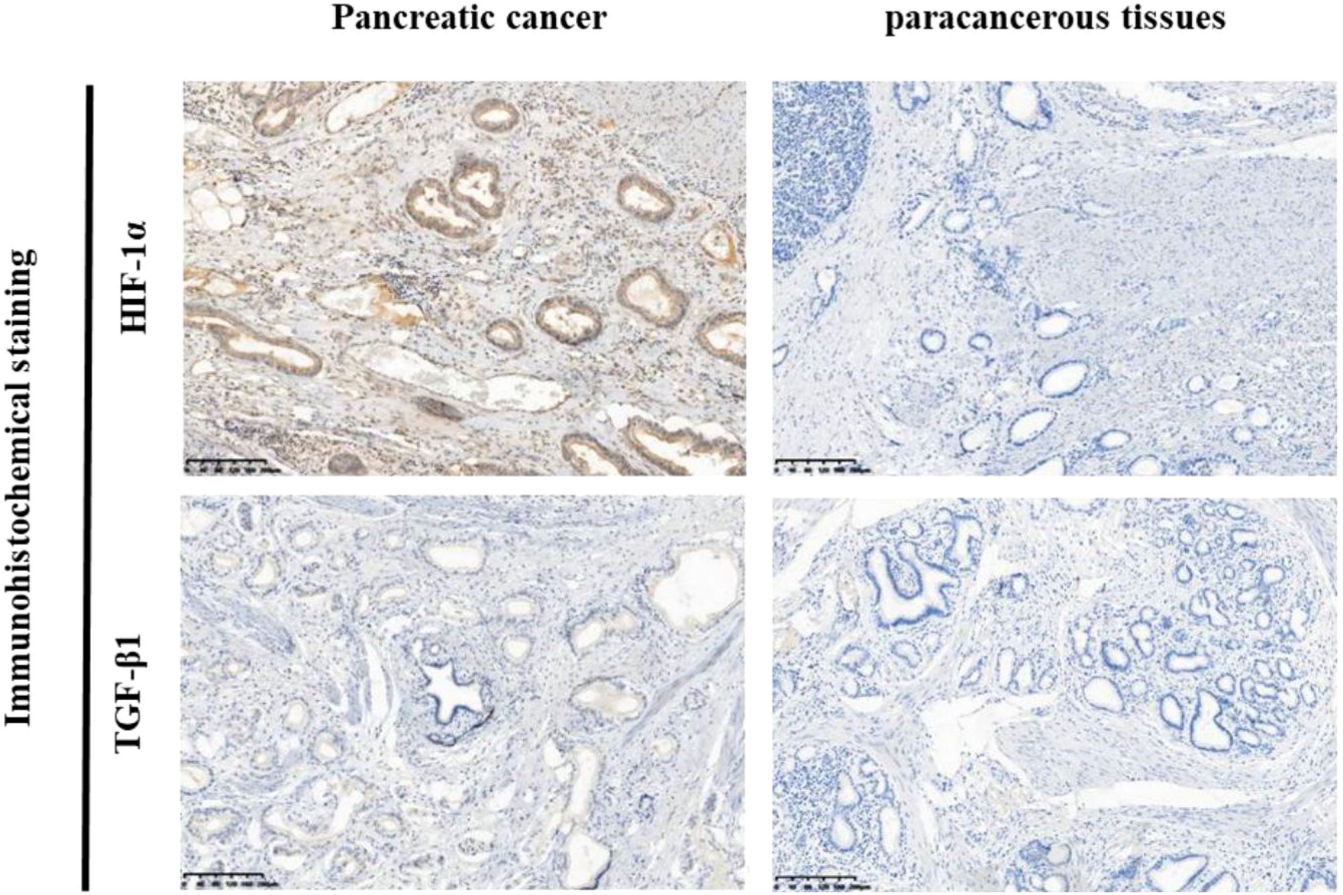
Immunohistochemical analysis of HIF-1α and TGF-β1 expression in pancreatic cancer tissues and adjacent normal tissues. Representative images of immunohistochemical staining for HIF-1α and TGF-β1 in pancreatic cancer tissues (left panel) and adjacent normal tissues (right panel). Positive staining is primarily localized in the cytoplasm (brown staining). Left side: High expression of HIF-1α and TGF-β1 in pancreatic cancer tissues. Right side: Low expression ofHIF-1α and TGF-β1 in adjacent normal tissues.

Immunohistochemical analysis revealed distinct expression patterns of HIF-1α and TGF-β1 in pancreatic cancer tissues compared to adjacent normal tissues. HIF-1α showed predominantly nuclear localization, while TGF-β1 exhibited cytoplasmic staining (**Figure 1**). Quantitative analysis demonstrated high HIF-1α expression in 73.33% (22/30) of cancer tissues compared to 23.33% (7/30) of adjacent normal tissues (*P* < 0.001). Similarly, TGF-β1 showed high expression in 63.33% (19/30) of cancer tissues versus 26.67% (8/30) of adjacent normal tissues (*P* < 0.004) (**Figure 1**, **Table 2**).

**Table 2:** Expression of HIF-1α and TGF-β1 in pancreatic cancer tissues and normal tissues.

Group	sample	HIF-1α		TGF-β1	
		Low	High	Low	High
Pancreatic Cancer Tissues	30	8	22	11	19
Normal Tissues	30	23	7	22	8
X^2^		15.017		8.148	
*P* value		<0.001		0.004	

### Correlation of HIF-1α and TGF-β1 expression with clinical features

**Figure 2:**
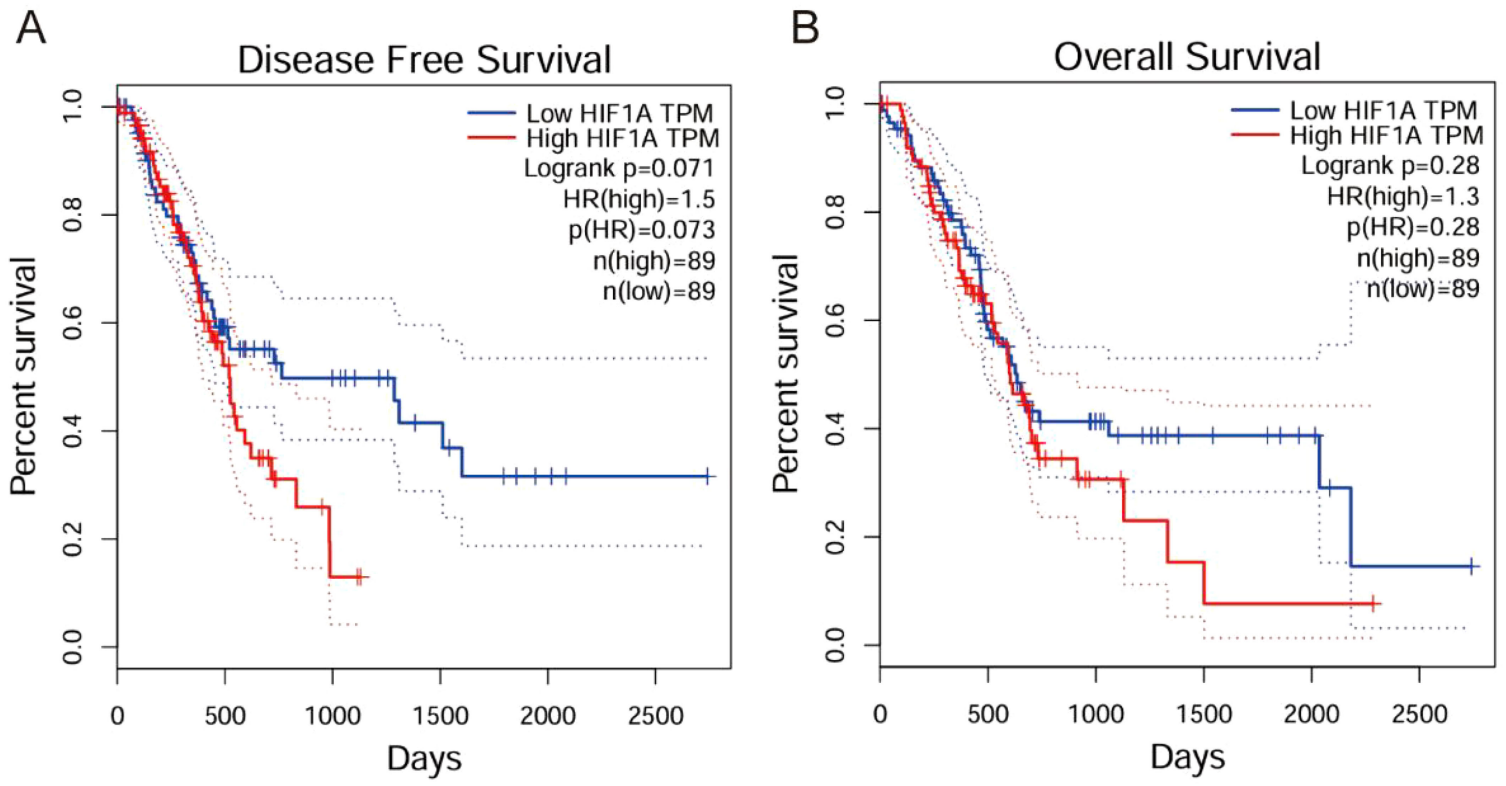
Kaplan-Meier survival analysis of HIF-1α in clinical practice. **(A)** Disease Free Survival analysis showing that high HIF1A expression correlates with poorer disease-free survival (Logrank p=0.071, HR(high)=1.5). **(B)** Overall Survival analysis demonstrating that high HIF1A expression correlates with reduced overall survival (Logrank p=0.28, HR(high)=1.3).

Analysis of clinical parameters revealed significant associations between HIF-1α/TGF-β1 expression and disease progression. Both factors showed higher expression in advanced TNM stages (III-IV) compared to early stages (I-II) (*P* < 0.020). Additionally, elevated expression correlated with lymph node metastasis (*P* < 0.018) (**Tables 3**, **4**). No significant correlations were observed with age, gender, tumor size, or tumor location. Furthermore, we conducted Kaplan-Meier survival analysis for HIF-1α, which revealed that high HIF-1A expression may promote tumor cell survival and metastasis in hypoxic microenvironments (**Figures 2A, B**).

**Table 3:** Relationship between HIF-1α expression and clinicopathological characteristics of pancreatic cancer patients.

Clinicopathological features	Sample (n=30)	HIF-1α		*P* value
		High (n=22)	Low (n=8)	
Sex				1.000
Male	17	12	5	
Female	13	10	3	
Ages				0.399
≤60	14	12	2	
>60	16	11	5	
Maximum diameter of tumor				1.000
≤4 cm	18	13	5	
>4 cm	12	9	3	
Tumor site				0.417
head	19	15	4	
tail	11	7	4	
TNM staging				0.020
I-II staging	13	9	4	
III-IV staging	17	13	4	
lymphatic node transfer				0.018
Yes	19	14	5	
No	11	8	3	

**Table 4:** Relationship between TGF-β1 expression and clinicopathological characteristics of pancreatic cancer patients.

Clinicopathological features	Sample (n=30)	TGF-β1		*P* value
		High (n=22)	Low (n=8)	
Sex				1.000
Male	17	14	3	
Female	13	9	4	
Ages				0.399
≤60	14	11	3	
>60	16	12	4	
Maximum diameter of tumor				1.000
≤4 cm	18	13	5	
>4 cm	12	10	2	
Tumor site				0.417
head	19	14	5	
tail	11	9	2	
TNM staging				0.020
I-II staging	13	11	2	
III-IV staging	17	12	5	
lymphatic node transfer				0.018
Yes	19	18	1	
No	11	5	6	

### HIF-1α and TGF-β1 cooperatively regulate ECM protein expression

**Figure 3:**
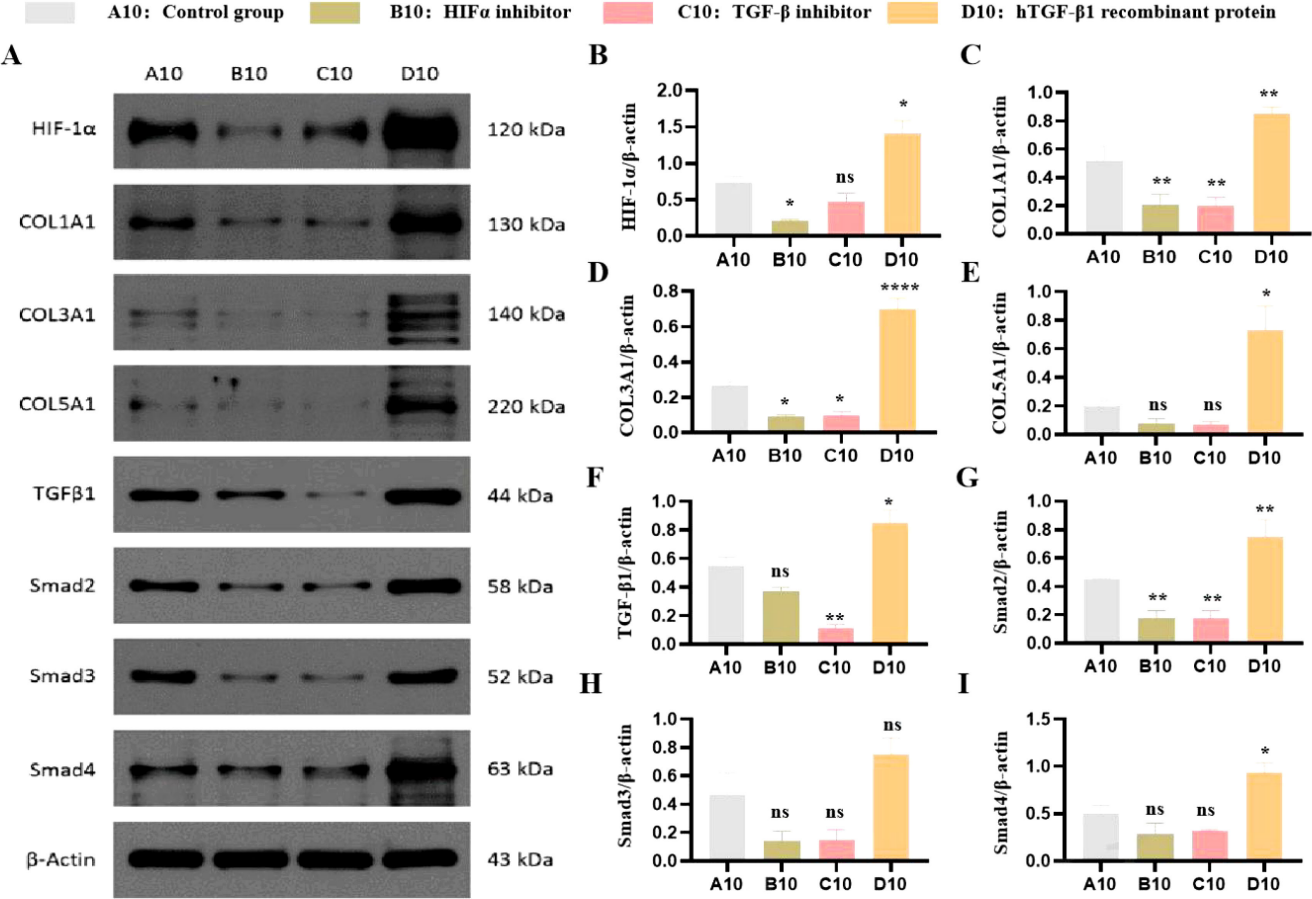
Effects of HIF-1α and TGF-β1 inhibitors and inducers on HIF-1α and collagen expression in PANC-1 cells. **(A)** Effects on HIF-1α Protein Expression. Western blot analysis showing the expression levels of HIF-1α protein in PANC-1 cells treated with a HIF-1α inhibitor (PX-478), a TGF-β1 receptor inhibitor (LY2157299), or hTGF-β1 recombinant protein compared to untreated controls. **(B)** Quantification of the western blot data showing the relative expression levels of HIF-1α. Data are presented as mean ± SEM from three independent experiments. **(C)** Effects on COL I Protein Expression. Western blot analysis showing the expression levels of COL I protein in PANC-1 cells treated with a HIF-1α inhibitor (PX-478), a TGF-β1 receptor inhibitor (LY2157299), or hTGF-β1 recombinant protein compared to untreated controls. **(D)** Quantification of the western blot data showing the relative expression levels of COL **(I)** Data are presented as mean ± SEM from three independent experiments. **(E)** Effects on COL III Protein Expression. Western blot analysis showing the expression levels of COL III protein in PANC-1 cells treated with a HIF-1α inhibitor (PX-478), a TGF-β1 receptor inhibitor (LY2157299), or hTGF-β1 recombinant protein compared to untreated controls. **(F)** Quantification of the western blot data showing the relative expression levels of COL III. Data are presented as mean ± SEM from three independent experiments. **(G)** Effects on COL V Protein Expression. Western blot analysis showing the expression levels of COL V protein in PANC-1 cells treated with a HIF-1α inhibitor (PX-478), a TGF-β1 receptor inhibitor (LY2157299), or hTGF-β1 recombinant protein compared to untreated controls. **(H)** Quantification of the western blot data showing the relative expression levels of COL V. Data are presented as mean ± SEM from three independent experiments. (* *P*<0.05; **p<0.01; ****p<0.0001; ns, no significant).

To investigate the functional relationship between HIF-1α and TGF-β1, we examined their effects on ECM protein expression under both normoxic and hypoxic conditions. Under normoxia, HIF-1α protein was detectable at baseline and increased significantly with TGF-β1 treatment. Hypoxia (1% O2) substantially enhanced HIF-1α protein levels and augmented TGF-β1-induced responses. The HIF-1α inhibitor PX-478 significantly reduced both basal and TGF-β1-stimulated expression of Collagen I, III, and V (*P* < 0.01). Similarly, the TGF-β1 receptor inhibitor LY2157299 decreased ECM protein expression. Conversely, recombinant TGF-β1 enhanced collagen expression, an effect that was more pronounced under hypoxic conditions (**Figures 3A–I**).

### HIF-1α is essential for TGF-β1-mediated ECM regulation

**Figure 4:**
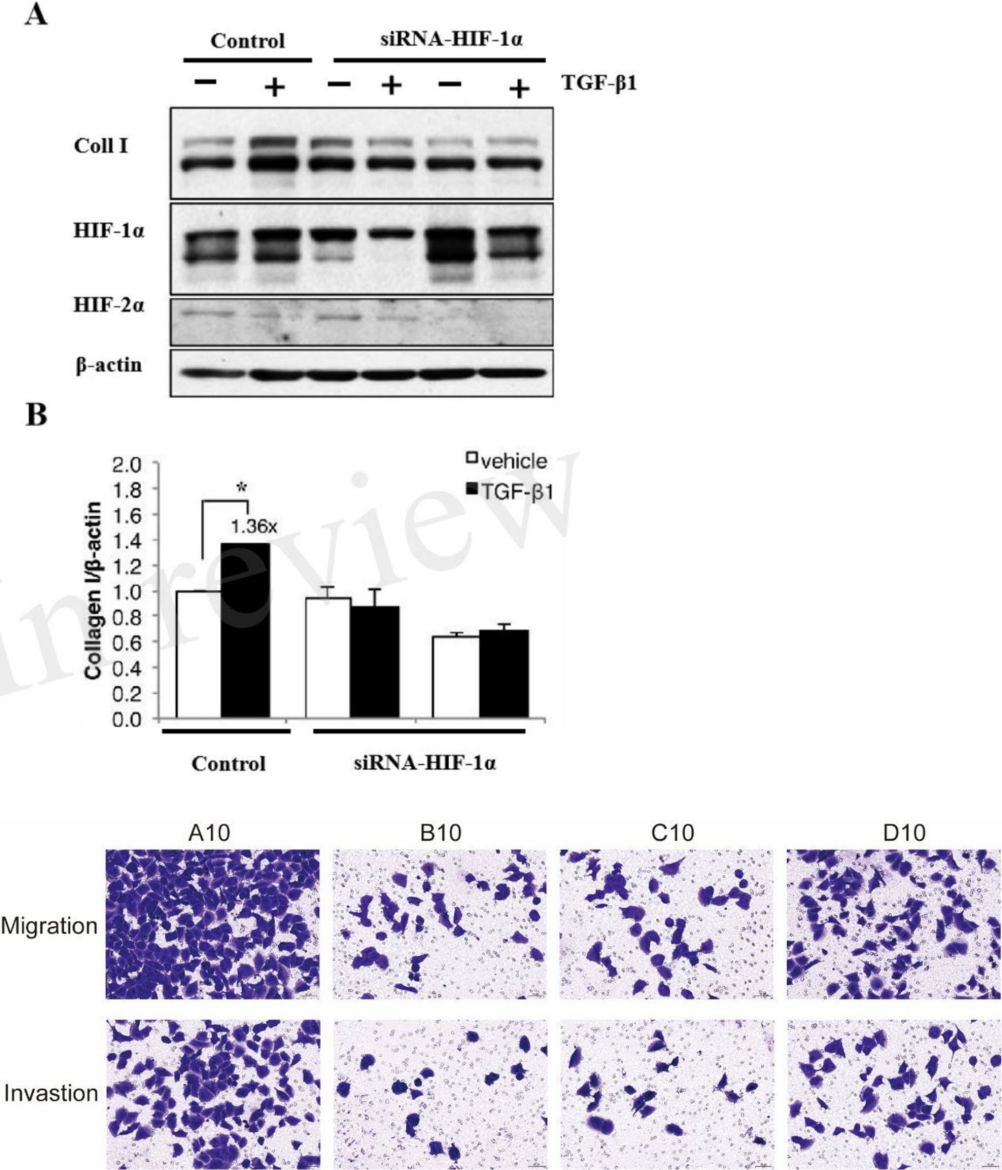
HIF-1α is essential for TGF-β1-mediated ECM regulation. **(A)** Western blot analysis showing the effect of HIF-1α knockdown on TGF-β1-induced collagen I expression in PANC-1 cells under normoxic and hypoxic conditions. **(B)** Quantitative analysis of collagen I protein expression relative to β-actin. Representative images of migration and invasion assays. A10: Control; B10: HIF-1α inhibitor (PX-478); C10: TGF-β1 receptor inhibitor (LY2157299); D10: hTGF-β1 recombinant protein. (* P<0.05).

To confirm the role of HIF-1α in TGF-β1 signaling, we performed siRNA-mediated knockdown of HIF-1α. HIF-1α depletion significantly attenuated TGF-β1-induced collagen expression under both normoxic and hypoxic conditions (**Figures 4A, B**). Western blot analysis confirmed successful knockdown of HIF-1α protein (>80% reduction). Furthermore, our migration and invasion assays demonstrated that inhibition of HIF-1α and TGF-β1 significantly suppressed both PANC-1 cell migration and invasion compared to the control group.

### Physical interaction between HIF-1α and TGF-β1 signaling components

**Figure 5:**
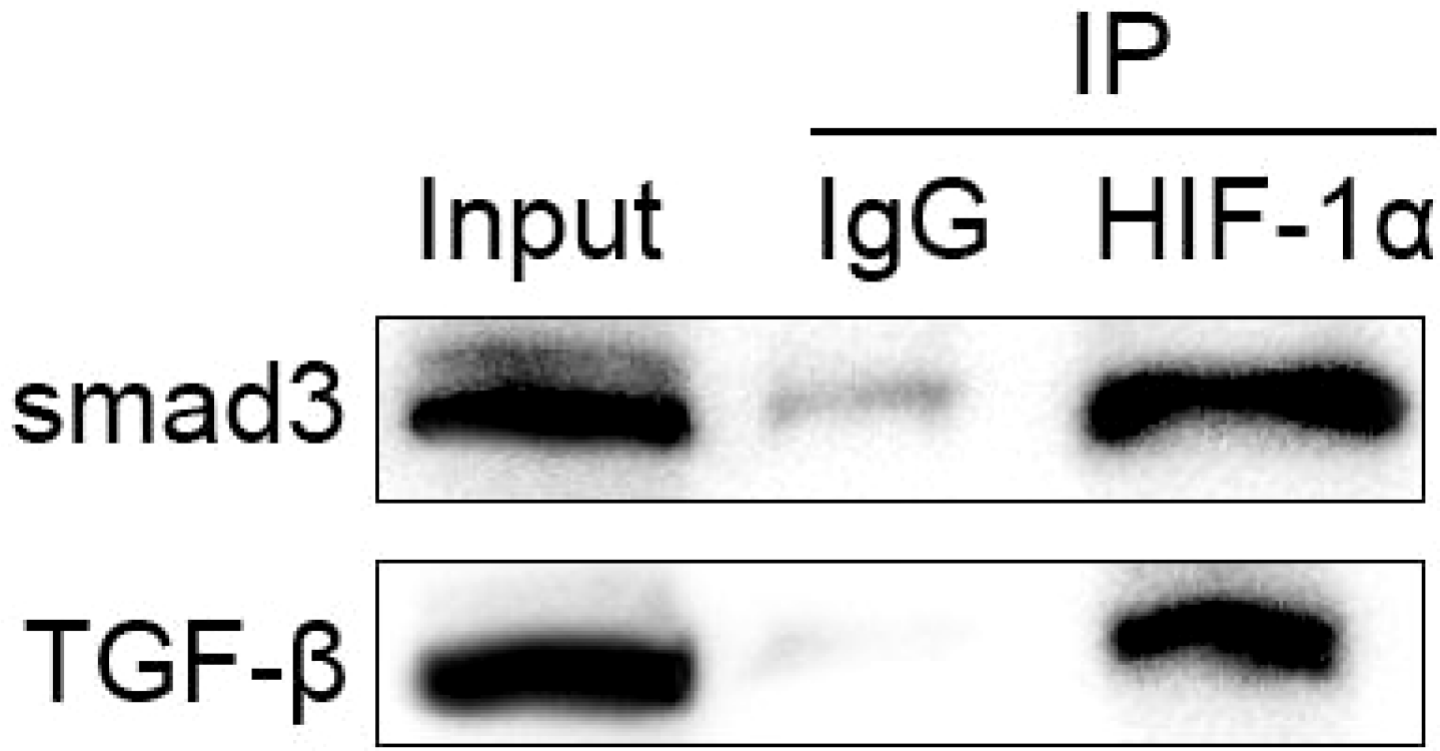
Physical interaction between HIF-1α and TGF-β1/Smad signaling components.

Co-immunoprecipitation experiments revealed a physical interaction between HIF-1α and components of the TGF-β1 signaling pathway. Moreover, immunoprecipitation with Smad3 antibody revealed that HIF-1α co-precipitated with Smad3, and their interaction was enhanced under hypoxic conditions (**Figure 5**). TGF-β1 stimulation further strengthened this association, indicating a direct molecular crosstalk between these pathways.

## Discussion

This study focuses on the specific role of the HIF-1α/TGF-β1/Smad axis in pancreatic cancer fibrosis, elucidating its mechanism of promoting tumor progression through ECM remodeling. Future research will validate combination therapies targeting this axis using preclinical models and explore stroma-based classification strategies to advance precision medicine in pancreatic cancer.

The fibrosis in pancreatic cancer is characterized by dense desmoplasia, where abnormal deposition of ECM components (e.g., collagen I, III, V) not only marks tumor progression but also acts as a critical barrier to therapeutic resistance (4). Our study found that HIF-1α and TGF-β1 are significantly overexpressed in pancreatic cancer tissues and collaboratively drive ECM protein synthesis via the Smad signaling pathway (**Figures 2**, **3**). This mechanism is closely linked to the pro-fibrotic microenvironment of pancreatic cancer. Clinically, high expression of HIF-1α and TGF-β1 correlates significantly with advanced TNM stages and lymph node metastasis (**Tables 3**, **4**), suggesting their potential as biomarkers for fibrosis and invasiveness. Furthermore, the dense stroma in pancreatic cancer restricts drug penetration through physical barriers while activating cancer-associated fibroblasts (CAFs) to secrete pro-fibrotic factors (e.g., TGF-β1), forming a vicious cycle (5). In this study, HIF-1α inhibitor (PX-478) and TGF-β receptor inhibitor (LY2157299) significantly reduced collagen expression (**Figure 2**), providing experimental evidence for disrupting this cycle.

TGF-β is a multifunctional cytokine regulating cell proliferation, differentiation, and migration (17). In models of diabetic nephropathy and glomerulosclerosis (18, 19), TGF-β increases collagen production, contributing to ECM remodeling and fibrosis (20). The TGF-β signaling cascade involves binding to type I (TβRI) and type II (TβRII) receptors, leading to phosphorylation of receptor-regulated Smads (R-Smads), such as Smad2 and Smad3 (21). These R-Smads then form a complex with Smad4 and translocate to the nucleus, where they regulate the transcription of TGF-β target genes, including collagen type I.

Beyond the canonical Smad pathway, TGF-β activates several non-Smad pathways, including p38, c-Jun N-terminal kinase (JNK), extracellular signal-regulated kinase (ERK) mitogen-activated protein kinases (MAPKs), and phosphoinositide 3-kinase (PI3K)-Akt-mammalian target of rapamycin (mTOR) signaling (22–24). These pathways play crucial roles in TGF-β-induced fibrosis. For instance, mTORC1 and mTORC2 are activated by TGF-β and are essential for its profibrotic effects on collagen synthesis.

HIF-1α has been shown to interact with the TGF-β signaling pathway, enhancing its profibrotic effects (25). HIF-1α modulates the expression of Smad molecules, thereby influencing TGF-β signaling. This interaction is particularly relevant under hypoxia, where HIF-1α activity is elevated, potentially amplifying TGF-β-mediated fibrosis. In pancreatic cancer, CAFs—a major source of TGF-β1—secrete ECM proteins that further activate HIF-1α, establishing a “hypoxia-TGF-β1-ECM” positive feedback loop (5). Targeting this loop may specifically destabilize stromal support in pancreatic cancer.

Our study reveals that HIF-1α and TGF-β1 cooperatively regulate pancreatic cancer ECM remodeling through direct interaction and Smad signaling, providing a foundation for therapeutic strategies targeting this axis. In this study, PX-478 effectively inhibited HIF-1α activity and significantly reduced TGF-β1-induced ECM protein (collagen I, III, V) expression (**Figure 3**). Preclinical studies indicate that HIF-1α inhibitors (e.g., PX-478, acriflavine) suppress tumor hypoxia adaptation and angiogenesis while enhancing chemosensitivity (9, 26). In combination chemotherapy, HIF-1α inhibition may alleviate hypoxic microenvironments in pancreatic cancer, improving the delivery efficiency of chemotherapeutic agents like gemcitabine (3). Given the central role of TGF-β in fibrosis, targeting this pathway offers promising therapeutic opportunities. Inhibiting TGF-β signaling via direct antagonists (e.g., TGF-β blockers) or indirect modulation of downstream pathways could mitigate fibrosis. In this study, LY2157299 (galunisertib) markedly suppressed TGF-β1 signaling and reduced ECM protein expression (**Figure 3**). Galunisertib has demonstrated antifibrotic and antitumor activity in pancreatic cancer clinical trials (NCT02734160), though its potential interference with TGF-β’s immunomodulatory functions requires caution (27). Similarly, Smad3—a key downstream effector of TGF-β1 signaling—can be targeted using oligonucleotide inhibitors (e.g., SIS3) or small molecules to block profibrotic signals, as validated in liver fibrosis models (28). Additional strategies targeting ECM remodeling include combining collagenase (e.g., MMP2/9 inhibitors) or hyaluronidase (PEGPH20) to disrupt dense stromal barriers and enhance drug penetration (4). TGF-β1 inhibition may also reverse the immunosuppressive phenotype of CAFs, potentially synergizing with PD-1/PD-L1 inhibitors to activate antitumor immunity (1). Therapeutic strategies targeting the HIF-1α/TGF-β1 axis hold significant promise in pancreatic cancer, but efficacy and safety require optimization through refined dosing regimens, selective inhibitors, and multi-target combinations. The mechanistic insights from this study provide a theoretical foundation, with future work focusing on *in vivo* validation and translational research.

While our findings offer valuable insights into the roles of HIF-1α and TGF-β in collagen regulation and fibrosis, this study has limitations. First, experiments were conducted *in vitro* using a single cell line; validation with multiple cell lines and *in vivo* models is necessary. Additionally, exploring the molecular mechanisms of HIF-1α/TGF-β interactions will deepen understanding of the regulatory pathways involved. Future studies should refine this mechanism through multi-cellular validation, animal experiments, large clinical cohorts, and functional analyses to strengthen the basis for targeted therapies.

In conclusion, our study highlights the importance of the HIF-1α-TGF-β axis in regulating collagen expression and fibrosis across organ systems. These findings underscore the potential of targeting these pathways to develop novel therapies that inhibit ECM remodeling and improve outcomes for patients with fibrotic diseases.

## Data Availability

The data presented in the study are deposited in the Figshare repository, accession number 28876760 (https://doi.org/10.6084/m9.figshare.28876760).
