# Correction: HIF‐1α modulates pancreatic cancer ECM proteins via the TGF‐β1/Smad signaling pathway introduction

**DOI:** 10.3389/fonc.2025.1648986

**Published:** 2025-07-02

**Authors:** Houle Guo, Zhanxue Zhao, Linxun Liu

**Affiliations:** ^1^ Suzhou Medical College of Soochow University, Suzhou, China; ^2^ Hepatological Surgery Department, Qinghai Provincial People’s Hospital, Xining, Qinghai, China

**Keywords:** transforming growth factor beta 1, hypoxia-inducible factor-1 alpha, extracellular matrix, Smad signaling pathway, pancreatic cancer

Affiliation assigned to the authors was incomplete. This affiliation has now been updated to “Hepatological Surgery Department, Qinghai Provincial People’s Hospital, Xining, Qinghai, China”.

Affiliation “Suzhou Medical College of Soochow University, Suzhou, China” was omitted from the paper. This affiliation has now been added for author Houle Guo.

The original version of this article has been updated.

