# Supplementary figures and images for: HIF‐1α modulates pancreatic cancer ECM proteins via the TGF‐β1/Smad signaling pathway introduction
Source: Front Oncol. 2025 May 8;15:1564655. doi: 10.3389/fonc.2025.1564655 (PMC12094911; doi:10.3389/fonc.2025.1564655)

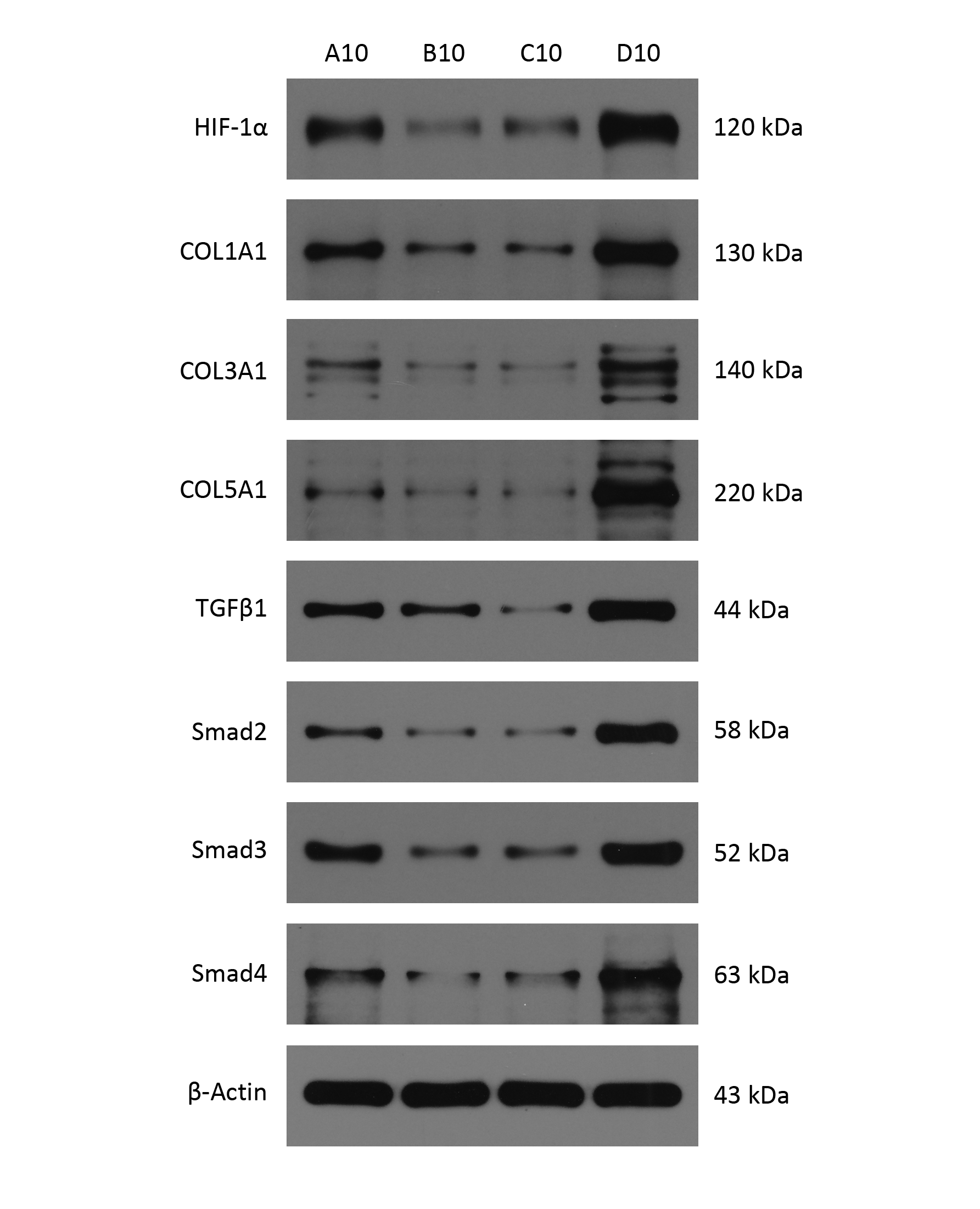

Supplement: Supplementary file 1 [file Image1.tif]

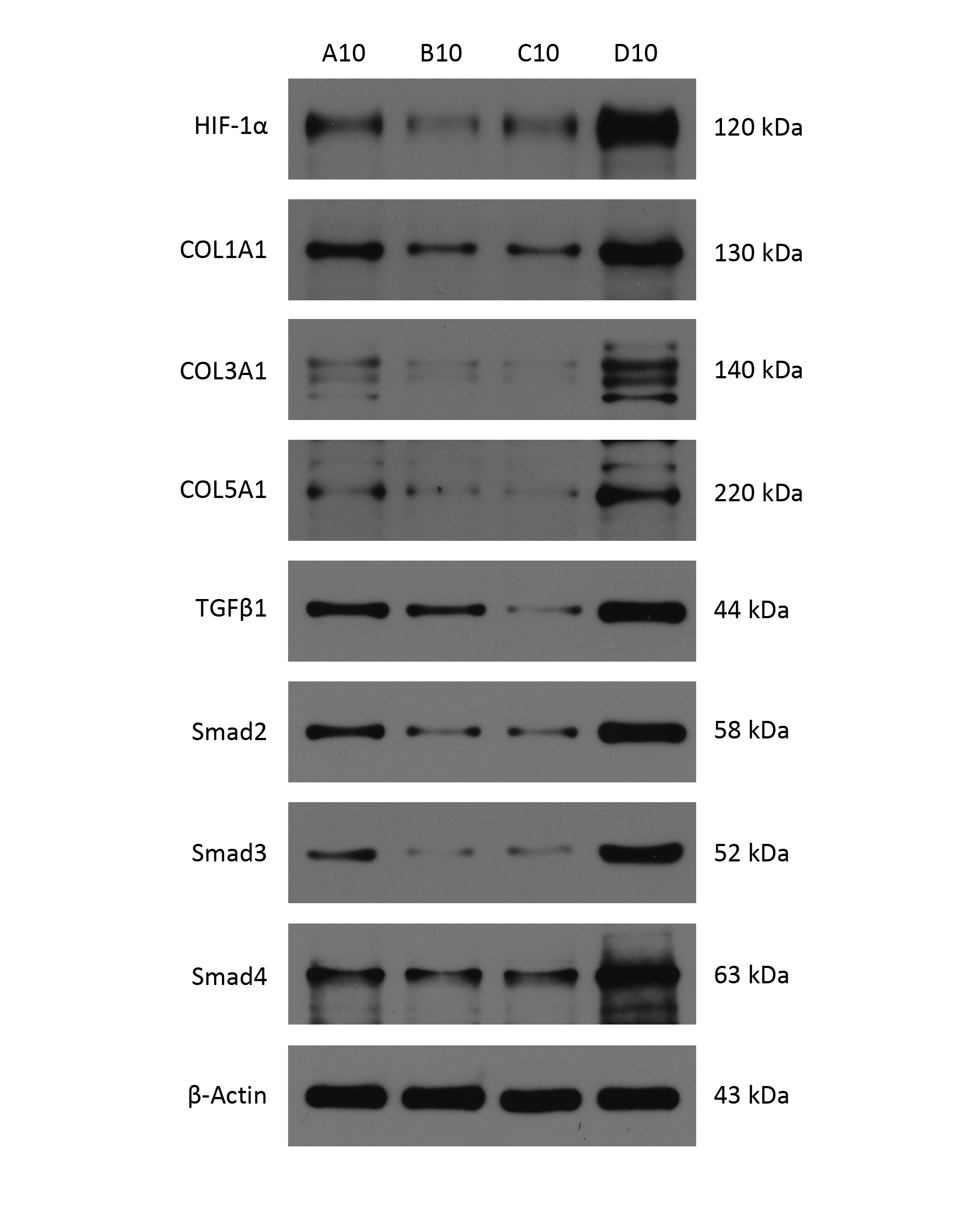

Supplement: Supplementary file 2 [file Image2.tif]

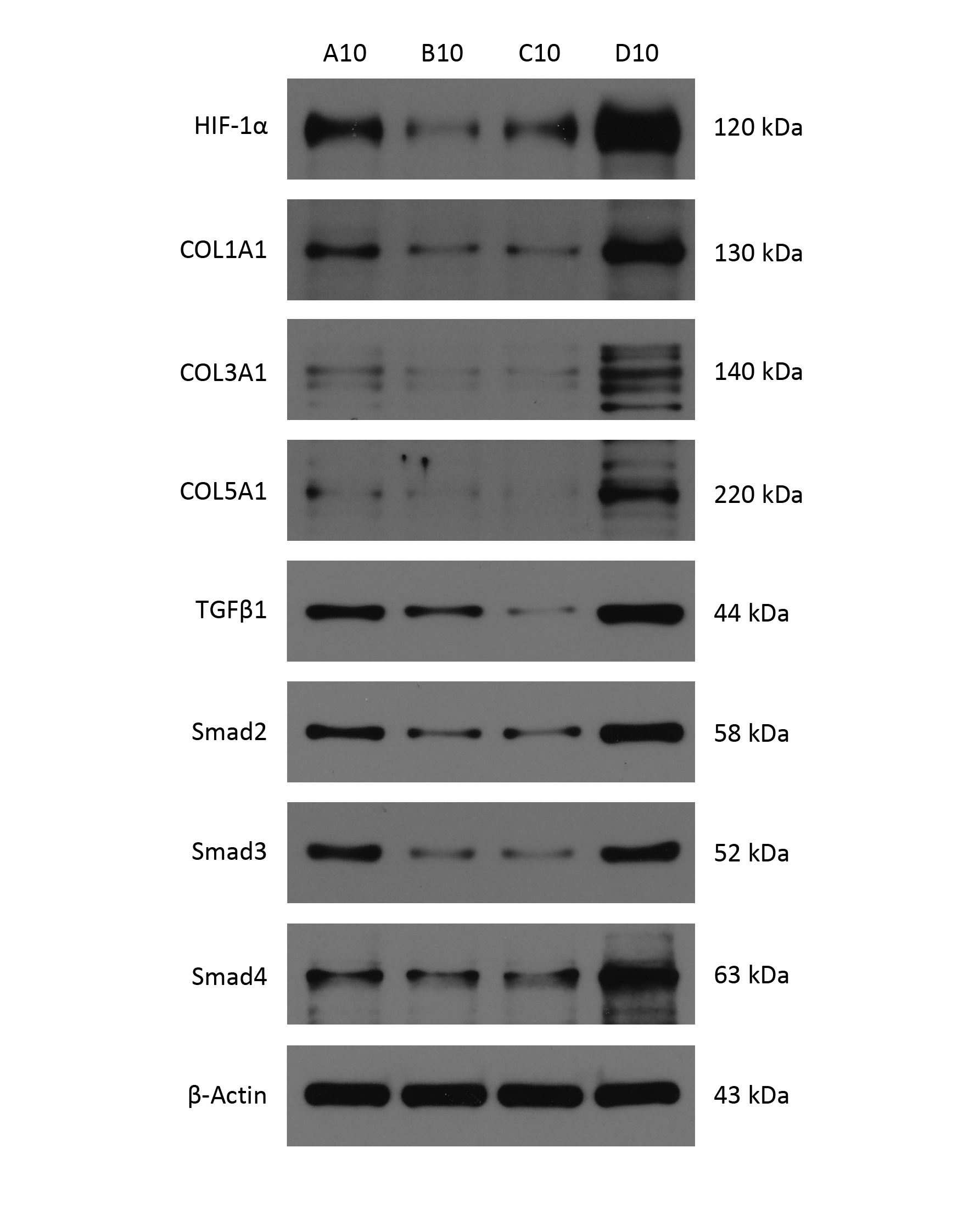

Supplement: Supplementary file 3 [file Image3.tif]
